# Mucous Stools in Infancy as an Early Marker of the Atopic March: A Four-Year Cohort Study of Respiratory Atopy Risk

**DOI:** 10.3390/children13020266

**Published:** 2026-02-13

**Authors:** Fatih Kaplan, Abdulgani Gülyüz

**Affiliations:** 1 Department of Pediatric Allergy and Immunology, Malatya Training and Research Hospital, Malatya 44000, Türkiye; fatih.kaplan@inonu.edu.tr; 2 Department of Pediatrics, Faculty of Medicine, Malatya Turgut Özal University, Malatya 44210, Türkiye

**Keywords:** mucous stools, food protein–induced allergic proctocolitis, atopic march, respiratory atopy, infant wheezing, allergic rhinitis, asthma, non–IgE-mediated food allergy, early-life immune priming, pediatric allergy

## Abstract

**Background:** Mucous stools in infancy are commonly attributed to non–IgE-mediated gastrointestinal food allergies and are generally considered transient and benign. However, whether mucous stools may indicate an atopy-prone clinical phenotype and relate to later respiratory atopy remains insufficiently explored. **Objective:** To evaluate the long-term risk of respiratory atopy (asthma and/or allergic rhinitis) in infants presenting with mucous stools during the first year of life and to identify early clinical predictors of this risk. **Methods:** This retrospective cohort study included infants who presented with mucous stools within the first 12 months of life and were followed for four years. Baseline demographic, clinical, dietary, and laboratory data were extracted from standardized medical records. Mucus severity was graded using a pragmatic 0–3 clinical mucus score. The primary outcome was physician-diagnosed asthma and/or allergic rhinitis at four years. Multivariable logistic regression was used to identify independent predictors, with model discrimination assessed by the area under the receiver operating characteristic curve (AUC). **Results:** A total of 142 infants with complete follow-up data were analyzed. At four years, respiratory atopy was observed in 45 infants (31.7%). In multivariable analysis, family history of atopy (adjusted odds ratio [aOR] 2.68, 95% CI 1.20–5.98, *p* = 0.016) and wheezing at presentation (aOR 3.74, 95% CI 1.56–8.94, *p* = 0.003) were independent predictors of respiratory atopy. The mucus score was associated with respiratory atopy in univariable analysis but did not remain an independent predictor in multivariable modeling. The model showed good discrimination (AUC = 0.769). **Conclusions:** In this cohort of infants presenting with mucous stools in the first year of life, respiratory atopy was observed in nearly one-third by age 4. While mucous stool burden was associated with the outcome in univariable analyses, it did not remain an independent predictor after adjustment. Early wheezing and a family history of atopy were the strongest clinical predictors and may help identify infants who warrant closer follow-up. These findings should be interpreted as associative and hypothesis-generating in the absence of a mucous-stool–free comparison group.

## 1. Introduction

Mucous stools are a common clinical finding in infancy and are most often evaluated within the spectrum of non–IgE-mediated food allergies, particularly food protein–induced allergic proctocolitis (FPIAP) [1,2,3,4]. Although this condition is generally considered to be limited to the gastrointestinal tract and transient in nature, in clinical practice many infants presenting with mucous stools simultaneously exhibit atopic features such as eczema, wheezing, or a family history of atopy. This observation suggests that mucous stools may represent an early clinical manifestation of a broader underlying immunological predisposition [3,4,5,6].

Although atopic diseases may manifest in different organ systems, they are generally regarded as dynamic expressions of a shared immunological background [7,8,9,10]. The concept of the “atopic march” describes the progression from early mucosal and cutaneous manifestations in infancy toward the later development of allergic diseases, particularly respiratory atopy [9,10]. Within this framework, gastrointestinal inflammatory findings such as mucous stools in early infancy are biologically plausible as early reflections of the systemic atopic predisposition that later gives rise to respiratory allergic diseases [7,8,11].

In the existing literature, mucous stools have predominantly been investigated in relation to short-term gastrointestinal outcomes, whereas their association with long-term respiratory atopic risk remains insufficiently explored [5,6]. In particular, it remains unclear whether the severity of mucous stools or the presence of concomitant early atopic manifestations can predict the development of respiratory atopic phenotypes later in life [1,6]. This gap suggests that infants presenting with mucous stools constitute a heterogeneous group, within which certain subgroups may carry a higher systemic atopic risk [5,6].

Early-life gastrointestinal mucosal inflammation has been linked to epithelial barrier dysfunction and immune activation that may contribute to later atopic disease in susceptible infants [7,8,11]. Accordingly, mucous stools may represent not merely a localized gastrointestinal symptom, but rather an early clinical manifestation of a systemic atopic predisposition [3,9,12].

The aim of this study was to evaluate the risk of developing respiratory atopy (asthma and/or allergic rhinitis) over a four-year follow-up period in infants presenting with mucous stools during the first year of life, and to determine whether early clinical indicators can predict this predisposition.

## 2. Materials and Methods

### 2.1. Study Design and Setting

This study is a retrospective, observational cohort study in which the clinical records of infants who presented with mucous stools within the first 12 months of life were reviewed and four-year clinical outcomes were evaluated. The study was conducted in the general pediatrics, pediatric allergy, and pediatric gastroenterology outpatient clinics of a tertiary-care university hospital. Baseline clinical and laboratory data were obtained from standardized assessment forms completed as part of routine clinical practice and from the electronic medical record system. Four-year clinical outcomes were retrospectively ascertained from outpatient follow-up records and the hospital information system.

### 2.2. Study Population

**Figure 1:**
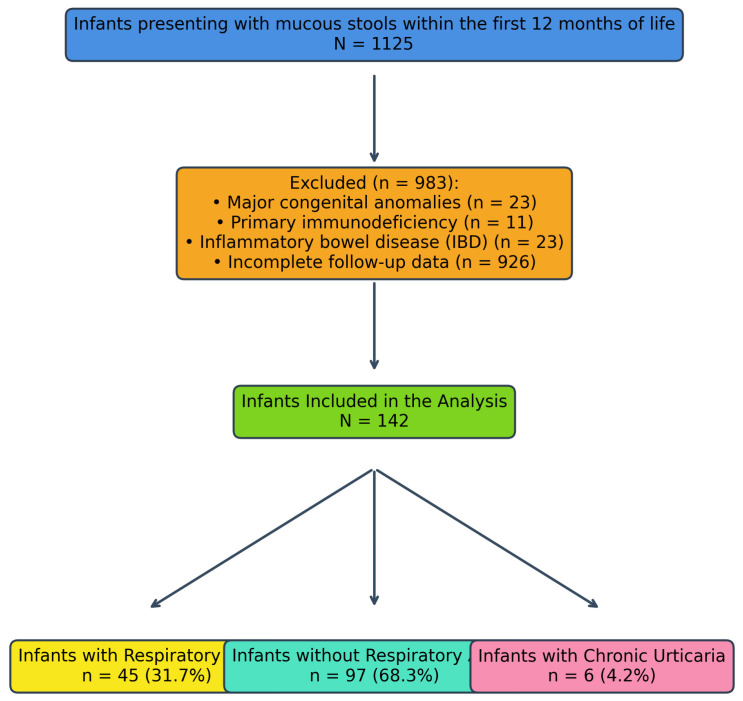
Patient flow diagram. Chronic urticaria is shown as a non–mutually exclusive exploratory outcome and does not represent a separate branching subgroup.

Infants who presented with macroscopic mucous stools within the first 12 months of life and who had no major congenital anomalies, primary immunodeficiency, inflammatory bowel disease, or organic gastrointestinal disease at baseline or during follow-up were eligible for inclusion. The presence of mucous in the stool was defined based on parental report at presentation and confirmation by the clinician through visible mucous on physical examination. All cases meeting the inclusion criteria within the specified time period were screened, and 142 infants with complete four-year outcome data constituted the final analytic cohort [Figure 1].

### 2.3. Baseline Assessment

At the initial presentation, demographic and perinatal characteristics, environmental factors (including antibiotic exposure and tobacco smoke exposure), feeding patterns, and family history of atopy were recorded. Laboratory assessments, including total IgE, specific IgE, skin prick testing, and peripheral blood eosinophil counts, were performed according to routine clinical practice protocols. The diagnosis of IgE-mediated food allergy was established by a pediatric allergist based on clinical history and test results in accordance with internationally accepted diagnostic criteria [13].

### 2.4. Mucus Score

The severity of mucous stools was graded at the initial presentation on a 0–3 scale (0 = none, 1 = mild, 2 = moderate, 3 = severe), based on parental report and clinician confirmation. The mucus score was not used as an objective biomarker but rather as a pragmatic, clinically based indicator of disease burden intended to reflect real-life pediatric practice [14]. Although not formally validated, this approach mirrors common bedside assessment in pediatric gastroenterology and allergy clinics and aims to capture clinically meaningful disease burden rather than laboratory-based severity metrics. The mucus score was not intended as a validated diagnostic or prognostic instrument but as a pragmatic, clinically oriented measure reflecting real-life pediatric practice. Formal assessment of inter-observer reliability was not performed, and this should be considered a methodological limitation. Accordingly, the mucus score was interpreted cautiously and evaluated primarily as a descriptive indicator of intestinal symptom burden rather than a definitive biomarker.

#### Outcome Definition and Diagnostic Ascertainment

The primary outcome was physician-diagnosed asthma and/or allergic rhinitis documented in routine outpatient follow-up records at the 4-year time point. Given the retrospective nature of the study, outcome diagnoses were based on the treating physician’s clinical judgment and standard clinical documentation rather than a uniform, protocol-driven diagnostic algorithm.

For asthma, diagnoses were recorded according to age-appropriate clinical criteria commonly used in pediatric practice, including recurrent wheezing episodes and response to bronchodilator therapy, recognizing that objective lung function testing is often not feasible or routinely performed at this age. Allergic rhinitis was defined based on persistent or recurrent rhinitis symptoms with clinical features consistent with allergic etiology, as documented by the treating physician. No requirement for mandatory spirometry or allergy testing was imposed for outcome definition.

### 2.5. Outcomes and Statistical Analysis

The primary outcome was defined as physician-diagnosed asthma and/or allergic rhinitis. After group comparisons, multivariable logistic regression was performed for the primary outcome. Covariates entered into the model (including age at presentation, family history of atopy, wheezing, mucus score, and eczema) were selected not solely based on statistical significance but also on biological plausibility and evidence from the literature. For secondary outcomes with low event numbers, Firth penalized logistic regression was used to reduce small-sample bias. Model discrimination was assessed using the area under the receiver operating characteristic (ROC) curve (AUC). Dietary interventions (elimination diet, probiotic use, and formula type) were tested in sensitivity analyses and did not materially alter effect estimates. Age refers to the age at initial presentation with mucous stools.

Covariates included in the multivariable logistic regression model were selected based not only on statistical associations but also on clinical applicability, biological plausibility from the literature, and the risk of multicollinearity. Although total IgE levels and eosinophil counts differed between groups, they were not included in the model in order to preserve bedside applicability without requiring additional laboratory testing. Moreover, these biomarkers did not provide clinically meaningful cut-off values capable of discriminating individual risk for respiratory atopy, and their distributions overlapped substantially between groups.

## 3. Results

### 3.1. Study Population

All 142 infants completed the four-year follow-up period and were included in the analyses of both primary and secondary outcomes. At baseline, 55.6% of the cohort were male, and the median age at presentation was 2 months (interquartile range [IQR], 1–3) (Table 1).

### 3.2. Four-Year Clinical Outcomes

At the four-year follow-up, asthma was identified in 20 infants (14.1%), allergic rhinitis in 34 infants (23.9%), and coexisting asthma and allergic rhinitis in 9 infants (6.3%). Overall, respiratory atopy (asthma and/or allergic rhinitis) was observed in 45 infants (31.7%). Chronic urticaria was detected in 6 infants (4.2%) and was evaluated as an exploratory outcome (Table 2).

### 3.3. Comparisons According to Baseline Characteristics

Infants who developed respiratory atopy had a higher prevalence of family history of atopy compared with those who did not (57.8% vs. 30.9%, *p* = 0.002), and wheezing was significantly more frequent in this group (42.2% vs. 17.5%, *p* = 0.002). The median mucus score was higher in the respiratory atopy group (*p* = 0.042); however, when mucus burden was dichotomized (scores 0–1 vs. 2–3), no significant difference was observed between groups (*p* = 0.135).

Total IgE levels and peripheral blood eosinophil counts were higher in the respiratory atopy group, but these parameters exhibited wide distributions and did not yield a clear discriminative cut-off between groups. There were no significant differences between infants with and without respiratory atopy with respect to maternal elimination diet, use of hypoallergenic formula, excluded foods (e.g., milk, egg, and other foods), probiotic use, or parental concern level (all *p* > 0.05). Likewise, no statistically significant differences were observed between groups for other demographic, perinatal, or environmental variables, including sex, prematurity, cesarean delivery, early antibiotic exposure, passive smoke exposure, visible blood in the stool, eczema, and poor weight gain (Table 1).

### 3.4. Regression Analysis for the Primary Outcome (Respiratory Atopy)

In univariable logistic regression analyses, wheezing (OR 3.44, 95% CI 1.56–7.58, *p* = 0.002), family history of atopy (OR 3.06, 95% CI 1.47–6.35, *p* = 0.003), and each one-point increase in the mucus score (OR 1.53, 95% CI 1.02–2.30, *p* = 0.039) were associated with respiratory atopy (Table 3).

In multivariable analysis, only family history of atopy (adjusted OR [aOR] 2.68, 95% CI 1.20–5.98, *p* = 0.016) and wheezing (aOR 3.74, 95% CI 1.56–8.94, *p* = 0.003) remained independent predictors of respiratory atopy. Mucus score, visible blood in stool, eczema, and IgE-mediated food allergy were not independently associated with the primary outcome in the multivariable model (Table 4). Total IgE levels, eosinophil counts, dietary factors, probiotic use, and parental concern were not retained in the final model because their inclusion did not materially change the results.

### 3.5. Secondary Outcomes

In multivariable analysis for asthma, age at presentation (aOR 1.81, *p* = 0.008) and wheezing (aOR 7.64, *p* < 0.001) were significant predictors. For allergic rhinitis, only family history of atopy was an independent determinant (aOR 3.25, *p* = 0.007), whereas the mucus score showed a borderline association (*p* = 0.063). In the penalized regression analysis for the severe phenotype defined by the coexistence of asthma and allergic rhinitis, both age at presentation (aOR 2.32, *p* = 0.020) and mucus score (aOR 6.20, *p* = 0.017) were associated with the outcome. Age at presentation was also associated with asthma and the combined asthma-and-rhinitis phenotype (Table 5). Given the limited number of events, these results should be interpreted as hypothesis-generating. For chronic urticaria, the number of events was too low to detect any meaningful associations with clinical, dietary, or laboratory variables.

## 4. Discussion

In this retrospective cohort study, approximately one-third of infants who presented with mucous stools within the first 12 months of life (31.7%, *n* = 45/142) were found to have developed respiratory atopy (asthma and/or allergic rhinitis) by the end of follow-up. The proportion of respiratory atopy observed among infants presenting with mucous stools was higher than that reported in previously published cohort studies, which may indicate a risk-enriched clinical phenotype; however, direct risk quantification versus infants without mucous stools was not possible in this design [15,16]. Therefore, the comparison should be interpreted as contextual rather than as a population-level risk estimate.

Although the mucus score was associated with the development of respiratory atopy in univariable analyses, this association lost independence in multivariable logistic regression, indicating that mucus burden likely reflects a shared underlying atopic diathesis captured by stronger clinical predictors such as wheezing and family history of atopy. The model demonstrated good discrimination (AUC = 0.769), with evidence of potential miscalibration (Hosmer–Lemeshow *p* = 0.015), indicating that predicted probabilities should be interpreted as relative risk stratification rather than absolute individual risk. In this context, the mucus score should be regarded not as a tool for generating precise individual probabilities, but rather as a pragmatic clinical marker for identifying infants at higher risk of an early atopy-prone clinical phenotype. Consistent with this interpretation, age at presentation emerged as a relevant factor for asthma-related secondary outcomes, but not for the primary outcome of respiratory atopy.

### 4.1. Atopic March and Immunological Mechanisms

Our findings are compatible with the atopic-march framework in the sense that infants presenting with mucous stools may represent a clinically risk-enriched subgroup in whom later respiratory atopy is more frequently observed [17,18,19,20,21]. However, the present retrospective design does not allow causal inference, and mucous stools should be considered a clinical correlate rather than proof of a mechanistic pathway.

Accordingly, a gastrointestinal-onset atopic phenotype should be regarded as a hypothesis-generating concept supported by associative clinical observations. Prospective studies incorporating objective biomarkers of intestinal barrier function and immune activation are needed to clarify whether early gastrointestinal inflammation contributes independently to subsequent respiratory atopy.

### 4.2. Analysis of Risk Factors

In multivariable analyses, family history of atopy (aOR 2.68, 95% CI 1.20–5.98, *p* = 0.016) and wheezing at presentation (aOR 3.74, 95% CI 1.56–8.94, *p* = 0.003) emerged as the strongest independent predictors of respiratory atopy. These findings are consistent with the established central role of early-life wheezing and genetic predisposition in asthma prediction models [22,23,24]. Although the mucus score was associated with respiratory atopy in univariable analyses, this association did not persist after adjustment for established clinical predictors in multivariable models. This finding suggests that mucus burden may reflect an underlying atopy-prone clinical context rather than acting as an independent determinant of future respiratory atopy. Therefore, the mucus score should be interpreted as a supportive clinical feature within a broader risk profile, rather than as a standalone predictive marker. In contrast, early wheezing and a family history of atopy emerged as the strongest independent predictors, highlighting their central role in early risk stratification [19].

The emergence of clinical phenotypic features (family history and wheezing) as stronger predictors than laboratory biomarkers underscores the sufficiency of a noninvasive and cost-effective approach to prognostic assessment in infants with mucous stools. The exclusion of total IgE and eosinophil counts from the multivariable model preserved its generalizability and bedside applicability across heterogeneous clinical settings. This reinforces the interpretation of mucous stools as a clinical signal within the atopic march rather than a laboratory-defined abnormality. The discrepancy between the ordinal and dichotomized mucus score analyses may indicate a graded association rather than a clear threshold effect; however, this observation should be interpreted cautiously.

### 4.3. Comparison with the Literature and Clinical Implications

In the existing literature, mucous stools have primarily been addressed within the framework of food allergy and allergic proctocolitis [15]. However, in our study, IgE-mediated food allergy did not independently predict respiratory atopy (aOR 0.95, *p* = 0.901), suggesting that this phenotype may belong to a broader non–IgE-mediated atopic spectrum rather than classical IgE-driven sensitization [11,12]. Clinically, these findings indicate that infants presenting with mucous stools should not be managed solely with dietary modification and symptom control, but should also be evaluated for future atopic risk [11,25,26,27,28]. In particular, infants with wheezing and a family history of atopy should be considered “high-risk atopic candidates” and followed with a multidisciplinary approach [22,23,24,29].

The lack of association between chronic urticaria and any clinical or biological variable suggests that the atopic risk signaled by mucous stools is linked to a respiratory-specific atopic pathway rather than to a generalized mast cell–mediated disease spectrum.

The retrospective design and single-center setting limit the generalizability of our findings; however, the observed effect sizes highlight the potential prognostic relevance of mucous stools in early life. These findings should be interpreted as associative and hypothesis-generating. Future prospective studies incorporating objective biomarkers of intestinal barrier function and immune activation are required to clarify whether early gastrointestinal inflammation contributes independently to subsequent respiratory atopy and to further delineate its role within the atopic march.

### 4.4. Strengths and Limitations

This study has several strengths, including a well-defined clinical cohort of infants presenting with mucous stools in early life, a standardized four-year follow-up period, and comprehensive assessment of clinically relevant outcomes using routinely available data. The focus on pragmatic clinical predictors enhances the real-world applicability of the findings.

Several limitations should also be acknowledged. The retrospective design precludes causal inference and limits control over potential confounders. The absence of a mucous-stool–free comparison group restricts direct risk quantification relative to the general population. Outcome diagnoses were based on routine clinical documentation, and diagnostic variability—particularly for asthma at preschool age—cannot be fully excluded. The mucus score represents a pragmatic, non-validated measure without formal assessment of inter-observer reliability and should therefore be interpreted cautiously. Finally, the limited number of events for certain secondary outcomes necessitates cautious interpretation of these analyses as hypothesis-generating. Because the analytic cohort was restricted to infants with complete four-year outcome documentation, selection or follow-up bias cannot be excluded, and infants with incomplete follow-up may have differed in baseline severity or referral patterns.

## 5. Conclusions

Among infants presenting with mucous stools in the first year of life, respiratory atopy was observed in nearly one-third by age 4. In adjusted analyses, early wheezing and a family history of atopy were the strongest independent predictors, whereas the mucus score did not retain independent predictive value. In the absence of a mucous-stool–free comparison group, these findings should be interpreted as associative and hypothesis-generating. Clinically, infants with mucous stools accompanied by wheezing or familial atopy may warrant closer follow-up for subsequent respiratory allergic disease.

## Figures and Tables

**Table 1:** Baseline characteristics of infants presenting with mucous stools (T0) according to the development of respiratory atopy (asthma and/or allergic rhinitis) at 4 years.

Variable	Total Cohort (*n* = 142)	Respiratory Atopy (+) (*n* = 45)	Respiratory Atopy (–) (*n* = 97)	*p*
Age at presentation, months	2 (1–3)	2 (1–3)	2 (1–3)	0.085
Male sex	79 (55.6)	29 (64.4)	50 (51.5)	0.150
Prematurity	9 (6.3)	1 (2.2)	8 (8.2)	0.272
Cesarean delivery	115 (81.0)	35 (77.8)	80 (82.5)	0.507
NICU admission	11 (7.7)	2 (4.4)	9 (9.3)	0.503
Early antibiotic exposure (0–30 days)	21 (14.8)	5 (11.1)	16 (16.5)	0.400
Passive smoke exposure	53 (37.3)	13 (28.9)	40 (41.2)	0.157
Family history of atopy	56 (39.4)	26 (57.8)	30 (30.9)	0.002
Mucus score (0–3)	2 (1–3)	2 (1–3)	2 (1–2)	0.042
High mucus burden (2–3)	85 (59.9)	31 (68.9)	54 (55.7)	0.135
Visible blood in stool	78 (54.9)	24 (53.3)	54 (55.7)	0.795
Atopic eczema	68 (47.9)	18 (40.0)	50 (51.5)	0.200
Wheezing	36 (25.4)	19 (42.2)	17 (17.5)	0.002
Poor weight gain	36 (25.4)	7 (15.6)	29 (29.9)	0.068
Concomitant IgE-mediated food allergy	47 (33.1)	15 (33.3)	32 (33.0)	0.968

Values are presented as *n* (%) or median (IQR). Groups were compared using the Mann–Whitney U test or chi-square/Fisher’s exact test. Family history of atopy was defined as asthma or allergic rhinitis in a first-degree relative. Percentages are column percentages.

**Table 2:** Four-year clinical outcomes in infants presenting with mucous stools.

Outcome	n/N	%	95% CI
Asthma	20/142	14.1	9.3–20.8
Allergic rhinitis	34/142	23.9	17.7–31.6
Respiratory atopy (asthma and/or allergic rhinitis)	45/142	31.7	24.6–39.7
Asthma AND rhinitis	9/142	6.3	3.4–11.6
Exploratory outcome: Chronic urticaria	6/142	4.2	2.0–8.9

Respiratory atopy was defined as physician-diagnosed asthma and/or allergic rhinitis at the four-year time point. Chronic urticaria was evaluated as an exploratory outcome.

**Table 3:** Univariable logistic regression for the primary outcome (4-year respiratory atopy).

Variable	OR (95% CI)	*p*
Wheezing	3.44 (1.56–7.58)	0.002
Family history of atopy	3.06 (1.47–6.35)	0.003
Mucus score (per +1 point)	1.53 (1.02–2.30)	0.039
High mucus burden (2–3)	1.76 (0.83–3.72)	0.137
Age at presentation (months)	1.30 (0.98–1.74)	0.072
Poor weight gain	0.49 (0.20–1.19)	0.116

OR indicates odds ratio; CI, confidence interval.

**Table 4:** Multivariable logistic regression for 4-year respiratory atopy (mucus score entered as an ordinal variable).

Variable	aOR (95% CI)	*p*
Age at presentation (months)	1.33 (0.96–1.84)	0.082
Family history of atopy	2.68 (1.20–5.98)	0.016
Wheezing	3.74 (1.56–8.94)	0.003
Mucus score (0–3)	1.36 (0.85–2.18)	0.200
Visible blood in stool	0.94 (0.41–2.14)	0.886
Eczema	0.61 (0.26–1.43)	0.254
Concomitant IgE-mediated food allergy	0.95 (0.40–2.25)	0.901

Model discrimination: AUC = 0.769. Model calibration: Hosmer–Lemeshow goodness-of-fit test, *p* = 0.015. Explained variance: Nagelkerke R^2^ = 0.21.

**Table 5:** Multivariable logistic regression analyses for secondary respiratory outcomes.

Variable	Asthma aOR (95% CI)	*p*	Allergic Rhinitis aOR (95% CI)	*p*	Asthma and Rhinitis aOR (95% CI)	*p*
Age at presentation (months)	1.81 (1.16–2.83)	0.008	–	–	2.32 (1.14–4.72)	0.020
Family history of atopy	–	–	3.25 (1.38–7.67)	0.007	–	–
Wheezing	7.64 (3.01–19.4)	<0.001	–	–	–	–
Mucus score (0–3)	1.49 (0.72–3.10)	0.286	1.64 (0.97–2.78)	0.063	6.20 (1.39–27.6)	0.017

Standard logistic regression was used for asthma and allergic rhinitis. Penalized (Firth) logistic regression was used for the asthma-and-rhinitis phenotype due to the limited number of events and to reduce small-sample bias. aOR indicates adjusted odds ratio; CI, confidence interval. Models were constructed based on clinically and biologically plausible variables.

## Data Availability

The datasets generated and/or analyzed during the current study are available from the corresponding author upon reasonable request.
